# WLLP: A weighted reconstruction-based linear label propagation algorithm for predicting potential therapeutic agents for COVID-19

**DOI:** 10.3389/fmicb.2022.1040252

**Published:** 2022-11-17

**Authors:** Langcheng Chen, Dongying Lin, Haojie Xu, Jianming Li, Lieqing Lin

**Affiliations:** ^1^Center of Campus Network and Modern Educational Technology, Guangdong University of Technology, Guangzhou, China; ^2^School of Computer Science, Guangdong University of Technology, Guangzhou, China

**Keywords:** COVID-19, drug repositioning, linear neighborhood similarity, label propagation, WKNKN

## Abstract

The global coronavirus disease 2019 (COVID-19) pandemic caused by the severe acute respiratory syndrome coronavirus-2 (SARS-CoV) has led to a huge health and economic crises. However, the research required to develop new drugs and vaccines is very expensive in terms of labor, money, and time. Owing to recent advances in data science, drug-repositioning technologies have become one of the most promising strategies available for developing effective treatment options. Using the previously reported human drug virus database (HDVD), we proposed a model to predict possible drug regimens based on a weighted reconstruction-based linear label propagation algorithm (WLLP). For the drug–virus association matrix, we used the weighted *K*-nearest known neighbors method for preprocessing and label propagation of the network based on the linear neighborhood similarity of drugs and viruses to obtain the final prediction results. In the framework of 10 times 10-fold cross-validated area under the receiver operating characteristic (ROC) curve (AUC), WLLP exhibited excellent performance with an AUC of 0.8828 ± 0.0037 and an area under the precision-recall curve of 0.5277 ± 0.0053, outperforming the other four models used for comparison. We also predicted effective drug regimens against SARS-CoV-2, and this case study showed that WLLP can be used to suggest potential drugs for the treatment of COVID-19.

## 1. Introduction

In November 2019, a novel coronavirus disease broke out in Wuhan, China, for unknown reasons, which was named coronavirus disease 2019 (COVID-19) by the World Health Organization (WHO) (Zhu et al., 2020). COVID-19 is caused by severe acute respiratory syndrome coronavirus-2 (SARS-CoV-2). To date, seven human coronaviruses (HCoV) have been identified, namely HCoV-229E, HCoV-OC43, HCoV-NL63, HCoV-HKU1, SARS-CoV, Middle East respiratory syndrome (MERS) coronavirus (MERS-CoV), and SARS-CoV-2. Specifically, HCoV-229E, HCoV-OC43, HCoV-NL63, and HCoV-HKU1 are frequently found and have low pathogenicity, generally causing only common cold symptoms, whereas MERS-CoV and SARS-CoV are zoonotic viruses that are first reported in the twenty-first century (Sohrabi et al., 2020). SARS-CoV-2 is recognized as the most pathogenic human coronavirus ever discovered (Guan et al., 2020). As of September 2022, 613 million confirmed SARS-CoV-2 infections were reported around the world, with nearly 6 million deaths (Organization, 2020). Until now, there is no cure for COVID-19.

Despite substantial increases in investment by pharmaceutical companies in response to COVID-19, the successful development and approval of a new drug typically requires billions of dollars and an average of 10 years (Liu S. et al., 2020), with the disadvantages of being time consuming (Pushpakom et al., 2019), expensive, and risky. Therefore, drug repositioning (drug repurposing) has been identified as a viable solution to improve the overall process of drug development, especially following recent advances in information technology and data science. The primary goal of drug repositioning is the use of existing drugs to treat new symptoms. Compared with traditional drug development methods, drug repositioning can significantly reduce research and development time and costs while minimizing risks. In short, drug repositioning is considered a promising strategy to accelerate the development of COVID-19 therapeutics.

As Xue et al. (2018) described, current work on drug repositioning is supported by various prediction models, among which the association prediction models for computational drug repositioning applicable to COVID-19 can be broadly classified into the following three categories (Dotolo et al., 2021): (I) network-based models, (II) artificial intelligence algorithms, and (III) matrix completion.

Network-based approaches construct heterogeneous networks by integrating multiple data to predict drug–virus associations. Such approaches are mostly based on the assumption that drugs with similar functions are often associated with viruses having similar phenotypes (Chen et al., 2018b). Prediction approaches based on complex networks (Liu et al., 2022b) have important and widespread applications in drug repositioning because of their ability to integrate multiple datasets of interest (Fan et al., 2020; Zhou et al., 2020). More specifically, network nodes represent drugs, diseases, viruses, or genes, while edges represent interactions or relationships between nodes (Re and Valentini, 2013; Chen et al., 2015). The obtained predictions may contribute to the process of structure-directed drug and diagnostic research and help to identify new potential biological targets (Barlow et al., 2020). In this regard, there are two network-based approaches applicable to drug repositioning for COVID-19: the network-based clustering approach and the network-based propagation approach. Macropol et al. (2009) proposed the repeated random walks (RRW) method that uses RRW on the protein–protein interaction (PPI) network for local clustering of the network and then predicts some protein complexes. Although this was found to be a precise and general approach, it requires a great deal of time and memory overhead and cannot detect overlapping clusters. King et al. (2012) introduced a new model named restricted neighborhood search clustering (RNSC), which is a global network algorithm for identifying protein clusters on PPI networks. It considers both global and local information from the network and can also detect overlapping clusters, but some information may be lost if the cluster size is too small. Luo et al. (2016) proposed the bidirectional random walk (BiRW) algorithm for predicting relationships between diseases and drugs. It uses the similarity of diseases and drugs with the original correlation matrix to form a heterogeneous network and then clusters this network by a double random walk. The resulting prediction is accurate, but more biological information is needed to improve the confidence of the similarity metric. In addition to the network clustering approach, Vanunu et al. (2010) proposed an overall propagation algorithm called PRINCE, which combines weighted PPI and disease similarity networks for overall disease gene ranking and protein complex association inference. An integrated propagation method for predicting propagation strategies in different sub-networks was proposed by Martinez et al. (2015) and named DrugNet. Zhang et al. (2017b) developed the linear neighborhood similarity (LNS) method to calculate drug–drug similarities in the drug characteristic space. Peng et al. (2021), in response to COVID-19, combined the virus–drug association network topology and a random walk with restart method (VDA-RWR) to predict potential drug–virus associations using a 2 × 2 similarity matrix and known associations between drugs and viruses. Zhang et al. (2021b) developed a network distance analysis model for the prediction of lncRNA–miRNA association (NDALMA). It is worth mentioning that the primary approach in recent years has been to update the network mainly by similarity and network inference (Zhang et al., 2021a; Liu et al., 2022a).

For drug repositioning, artificial intelligence-based models mainly use machine learning methods. Numerous common machine learning algorithms have been applied to predict potential therapeutic agents, such as decision trees (Chen et al., 2019b) and Laplacian regularization (Chen and Huang, 2017). The influence of deep learning models that belong to machine learning has been particularly remarkable (Chen et al., 2019a, 2021a; Keshavarzi Arshadi et al., 2020). In terms of prediction, graph convolutional neural networks (GCNNs) are the most popular tools for drug discovery applications because they can process graphs and extract features by encoding adjacency information within features to learn representations from molecules. Based on drug-target interactions in this model, Torng and Altman (2019) made correlation predictions. In recent years, sequence-based models, such as genomics, proteomics, and transcriptomics, have also attracted considerable attention. Vaswani et al. (2017) and Devlin et al. (2018) advanced a transformer model for extracting features from sequences through the attention mechanism and self-supervision, which are widely used in the field of natural language processing. Moreover, Shin et al. (2019) demonstrated that drug-target interactions can be predicted by using the transformer model. Pollastri et al. (2002) demonstrated that recurrent neural networks (RNNs) and long short-term memory (LSTM) networks can predict the secondary structure of molecular or protein sequences. Through an ensemble strategy of three mainstream machine learning algorithms, Hu et al. (2018) proposed a model named HLPI-Ensemble that was specifically designed for human lncRNA–protein interactions. Matrix completion mainly relies on the matrix decomposition algorithms (Chen et al., 2018a,c). Specifically, these algorithms decompose a matrix into two lower-order potential factor matrices based on known association matrices of diseases and drugs (Liu H. et al., 2020). Gönen (Gönen, 2012) put forward a prediction method by using Bayesian probabilistic matrix factorization (BPMF) based on chemical and nuclear genomes. Yang et al. (2019) developed a model based on bounded nuclear norm regularization for drug repositioning. Considering the similarity information between drugs and diseases, Meng et al. (2021) proposed a method called similarity-constrained PMF (SCPMF) to examine the potential value of existing drugs. Liu et al. (2022b) proposed a new computational method *via* deep forest ensemble learning based on an autoencoder (DFELMDA) to predict miRNA–disease associations.

The novel similarity measure of LNS proposed by Zhang et al. has been successfully applied to several bioinformatics problems (Zhang et al., 2017a, 2018a). In this method, the data points are reconstructed by linear neighborhood information and are used to measure the similarity between two points in the association network. Inspired by this, we applied this similarity measure to our model. In recent years, label propagation has been widely used for biological association prediction owing to its various advantages, such as simple logic algorithm and fast optimization. Thus, we adopted the label propagation method for network propagation of the drug–virus association matrix.

Herein, we reported on the development of a method termed label propagation through linear neighborhood similarity for the prediction of undetected drug–virus associations. More specifically, we represented drugs or viruses as feature vectors and treated them as data points in the feature space, from which we computed pairwise linear neighborhood similarities between drugs and drugs or between viruses and viruses. The computed drug and virus similarities and the known disease–virus association networks were treated as a weighted directed graph, which was then input to the label propagation algorithm. Each drug–virus interaction was scored using the label propagation method. Experiments showed that the WLLP model offered superior prediction results when compared with other models, with an area under the receiver operating characteristic (ROC) curve (AUC) of 0.8828 in the framework of 10 times 10-fold cross-validated.

## 2. Materials and methods

### 2.1. Experimental data

#### 2.1.1. Human drug virus database

The collection of data concerning viruses, drugs, and drug–virus associations is a crucial precursor to using bioinformatics methods to predict novel drug–virus associations. Moreover, systematic collection and management of relevant information are important for further studying the mechanism of virus action (Wang et al., 2021). Meng et al. (2021) collected a large number of experimentally validated drug–virus interaction entries from the literature by using text mining techniques and then constructed the HDVD, which is a database of human drug–virus associations. The HDVD includes 34 viruses, 219 drugs, and 455 confirmed human drug–virus interactions.

#### 2.1.2. Construction of the drug–virus interaction network

From the HDVD dataset, we constructed an association network using known drug–virus interactions, where the points represent the drugs and viruses and the edges represent drug–virus associations. Let *G* = (*D, V, I*) represents the drug–virus association network, where *D* = {*d*_1_, *d*_2_, …, *d*_*n*_} represents the known drugs in the dataset, *V* = {*v*_1_, *v*_2_, …, *v*_*m*_} represents the known viruses in the dataset, and *I* represents the interaction relationship between *D* and *V*. Let *A*_*n*×*m*_ represents the adjacency matrix of graph *G*. If *d*_*i*_ and *v*_*j*_ are related, *A*_*ij*_ = 1; otherwise, *A*_*ij*_ = 0. Also, let *A*^*T*^ represent the inversion of *A*_*n*×*m*_.

#### 2.1.3. Chemical structure similarity of drug pairs

The chemical structure similarity between two drugs can be calculated from their molecular structure information. In the current study, we downloaded the chemical structure information of drugs from the DrugBank database in the SMILES format (Öztürk et al., 2016) and then calculated their molecular access system (MACCS) fingerprints (O'Boyle et al., 2011). Finally, we used the Tanimoto index to measure the absolute similarity between two molecules (Bajusz et al., 2015). Specifically, we set two drug molecules as A and B, respectively, a is the number of bits in molecule A, and b is number of bits in molecule B. c is the number of bits that are in both molecules. The formula is as follows:


(1) $$\begin{array}{l} T=c/\left(a+b-c\right) \end{array}$$

We used this formula to construct the drug chemical structure similarity matrix *DD*_*n*×*n*_. This is a two-dimensional matrix whose values represent the chemical fingerprint scores between drugs. In general, the size of this score is between 0 and 1, with larger values representing greater drug–drug similarity.

#### 2.1.4. Genomic sequence similarity of virus pairs

The sequence similarity between viruses can be calculated from their genomic nucleotide sequences. We downloaded the genomic nucleotide sequences of viruses from the National Center for Biotechnology Information (Wheeler et al., 2002). To calculate the sequence similarity, we used the multiple sequence alignment program MAFFT on account of its high performance (Katoh and Standley, 2013). Finally, the virus sequence similarity matrix *VV*_*m*×*m*_ was constructed, which is a two-dimensional matrix whose values represent the sequence similarity between viruses. In general, the value of this matrix is between 0 and 1, and larger values represent greater virus–virus similarity.

### 2.2. Methods

#### 2.2.1. Overview of WLLP

**Figure 1:**
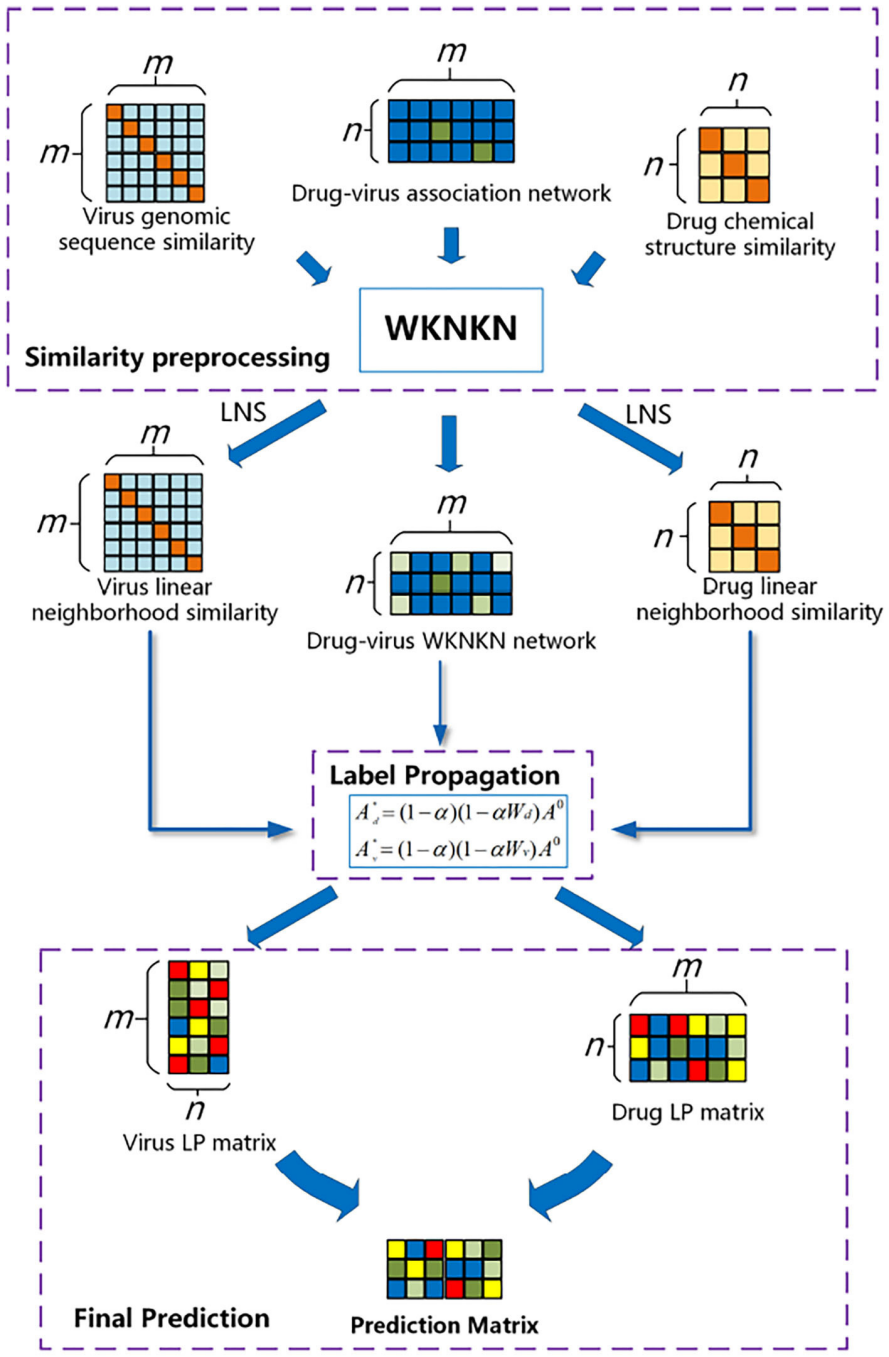
Flowchart of the weighted reconstruction-based linear label propagation algorithm (WLLP) framework for drug–virus association prediction.

In this study, we developed the WLLP framework for predicting disease–virus associations based on LNS in conjunction with label propagation. As shown in Figure 1, the framework consists of three main steps: (I) Label set preprocessing: considering the sparse nature of the drug–virus interaction matrix, we introduced the weighted K-nearest known neighbors (WKNKN) algorithm to make a correction for the potential interactions between the drugs and viruses. (II) LNS information for the drugs and viruses was mined separately based on drug–virus interaction information. (III) Label propagation: a weighted directed graph consisting of known association information, drug–drug LNS, and virus–virus LNS matrices was constructed, and the drug label information was iteratively updated by the label propagation algorithm to reveal unknown potential drug–virus associations.

The flowchart of the WLLP algorithm is shown in Algorithm 1. The details of the principle and process of each WLLP module are described in the following sections.

**Algorithm 1:** WLLP.

**Input:** Matrices *A*_*n*×*m*_, *DD*_*n*×*n*_, and *VV*_*m*×*m*_; Number of neighbors *K* and decay factor *r*; LNS size parameters *dN* and *DN*; Probability retention factors for drugs and viruses, α and β, respectively; **Output:** Predictive association matrix $A_{m\times n}^{*}$
1: Step 1: Reconstruct the association matrix
2: **for** *i* = 1 to *n* **do**
3: construct *A*_*d*_ using Equation (2)
4: **for** *i* = 1 to *n* **do**
5: construct *A*_*v*_ using Equation (3)
6: $A_{dv}=\left(A_{d}+A_{v}^{\text{T}}\right)/2$
7: *A* = *max*(*A, A*_*dv*_)
8: Step 2: Construct the drug LNS matrix *W*_*d*_
9: **for** *i* = 1 to *DN* **do**
10: construct refactoring weight *w*_*i*_ for each drug using Equation (5)
11: Step 3: Similarly, construct the virus LNS matrix *W*_*v*_
12: Step 4: Update the associated network by label propagation
13: Predict new association matrix $A_{d}^{*}$ using Equation (8) and weight *W*_*d*_
14: Similarly, predict the new association matrix $A_{v}^{*}$
15: $A^{*}=\left(A_{d}^{*}+A_{v}^{*\text{T}}\right)/2$
16: **return** A^*^

#### 2.2.2. WKNKN

Because it is hard to construct expression datasets, coming up with datasets that contain a large number of samples is generally difficult. A small number of samples complicates the knowledge discovery task (Sirin et al., 2016). The unknown nature of a large part of the information makes the drug–virus association matrix very sparse. Here, we used the WKNKN algorithm to preprocess the original association matrix (Ezzat et al., 2016). Specifically, WKNKN replaces *A*_0*ij*_=0 with the interaction likelihood in the following three steps:

**Step 1**. For each known drug, the chemical structure similarities of the closest *K* known drugs are calculated by the *k*-nearest neighbors (KNN) method and their corresponding interaction profiles are used to estimate the interaction likelihood profiles. The derived formula is


(2) $$\begin{array}{l} A_{d}\left(i,:\right)=\frac{\Sigma_{\text{k}=1}^{\text{K}}T^{k-1}DD\left(i,D_{k}\right)A\left(D_{k},:\right)}{\Sigma_{k=1}^{K}DD\left(i,D_{k}\right)}, \end{array}$$

where *i* denotes the drug index, *T* is the decay factor, and in general, *T* ≤ 1. *D*_*k*_ denotes the *k*-th drug index that is most similar to drug *i*. It is worthwhile to mention that the denominator part is the normalization term.

**Step 2**. For each known virus, the sequence similarities of the closest K known viruses are calculated by the KNN method and their corresponding interaction profiles are used to estimate the interaction likelihood profiles:


(3) $$\begin{array}{l} A_{v}\left(:,j\right)=\frac{\Sigma_{\text{k}=1}^{\text{K}}T^{k-1}VV\left(j,V_{k}\right)A^{T}\left(V_{k},:\right)}{\Sigma_{k=1}^{K}VV\left(j,V_{k}\right)}, \end{array}$$

where *j* denotes the virus index, *T* is the decay factor, and in general, *T* ≤ 1. *V*_*k*_ denotes the *k*-th virus index that is most similar to virus *j*. Similarly, the denominator part is the normalization term.

**Step 3**. If *A*_*ij*_ = 0, then we average the interaction likelihood values calculated by Equations (2) and (3) and replace the original values. Using WKNKN, we finally calculate a weighted nearest neighbor interaction spectrum, which we will substitute into the prediction model later.

#### 2.2.3. LNS

Previous studies have demonstrated that each data point can be perfectly reconstructed with linear neighborhood information (Wang and Zhang, 2006; Chen et al., 2021b). Based on these studies, we used the known drug–virus interactions to update the degree of drug–virus similarity. Inspired by Zhang et al. (2018b), we established linear neighborhood similarity. In the following, we analyzed the drugs as an example. We take the association matrix of drugs as *X* = {*x*_1_, *x*_2_, …, *x*_*n*_}, and each vector *x*_*i*_ is reconstructed from a linear combination of its neighboring data points. The objective function is to minimize the reconstruction loss with the following expression:


(4) $$\begin{array}{l} \underset{w_{i}}{\text{min}}L_{i}={\left|\left|x_{i}-\sum_{i_{j}:x_{i_{j}}\in N(x_{i})}w_{i,i_{j}}x_{i_{j}}\right|\right|}^{2}=\omega_{i}^{T}G^{i}\omega_{i}\\ s.t.\;\sum_{i_{j};x_{i_{j}}\in N(x_{i})}\omega_{i,i_{j}}x_{i_{j}}=1,\omega_{i}\geq 0,\text{j}=1,\ldots ,\text{DN}, \end{array}$$

where *N*(*x*_*i*_) denotes the set of *DN* nearest neighbors and *DN*(0 < *DN*<*n*) is a conditioning parameter that indicates the number of neighbors. *x*_*i*_*j*__ denotes the *j*-th neighbor of the vector *x*_*i*_. *w*_*i*_ = {*w*_*i*,_*i*__1__, *w*_*i*,_*i*__2__, …, *w*_*i*,_*i*__*DN*__}is a vector whose size is *DN*×1 representing the weight size of the *k* nearest neighbors of *x*_*i*_ and also indicates the similarity between *x*_*i*_*j*__ and *x*_*i*_. *G*_*i*_ denotes the gram matrix whose size is *DN*×*DN*, where $G_{i_{p},i_{q}}^{i}=\left(x_{i}-x_{i_{p}}\right){\left(x_{i}-x_{i_{q}}\right)}^{T}$. To prevent overfitting, we incorporated the Tikhonov regularization term, which makes the minimization reconstruction loss normalized. The formula is as follows:


(5) $$\begin{array}{l} \begin{array}{c} \underset{w_{i}}{\min}L_{i}=\omega_{i}^{\text{T}}G^{i}\omega_{i}+\mu ||\omega_{i}|{|}_{1}^{2}=\omega_{i}^{\text{T}}\left(G^{i}+\mu I\right)\omega_{i},\\ s.t.\underset{i_{j}:x_{i_{j}}\in N\left(x_{i}\right)}{\sum }\omega_{i,i_{j}}x_{i_{j}}=1,\omega_{i}\geq 0,j=1,\ldots ,\text{DN}, \end{array} \end{array}$$

where μ is the regularization factor. For simplicity, we set μ to 1. Finally, we used the standard quadratic programming method to solve the objective function, and the result can be regarded as the reconstruction weight of each data point *x*_*i*_. We thus obtained two weight matrices, $W_{d}\in R^{n\times n}$ and $W_{v}\in R^{m\times m}$, which were the LNS matrices for the drugs and viruses, respectively.

#### 2.2.4. Label propagation

From the previous calculation steps, we finally obtained three matrices: the drug–virus association matrix *A*_*n*×*m*_ after WKNKN processing, the drug–drug LNS matrix *W*_*d*_, and the virus–virus LNS matrix *W*_*v*_. In the following, as a representative example, we considered the drug–drug LNS matrix as a directed weighted graph, with drugs as the nodes and the degree of similarity as the weights of the lines. It is worth noting that the similarity matrix is not diagonally symmetric, i.e., *w*_*ij*_≠*w*_*ji*_. Based on this, we used a label propagation approach to circularly and iteratively propagate the label information of the drugs to reveal potential drug–virus associations. On the association network, the neighboring edge information of each drug was computed and updated at each label propagation. Meanwhile, we set a probability parameter α to retain its updated state and retain its initial state with a probability of 1−α. The specific updated equation is as follows:


(6) $$\begin{array}{l} A_{j}^{t+1}=\alpha W_{d}A_{j}^{t}+\left(1-\alpha \right)A_{j}^{0} \end{array}$$

where, for the exact virus *v*_*j*_, $A_{j}^{0}$ denotes all known original drug interaction relationships and $A_{j}^{t}$ denotes the predicted label at iteration *t*. For all viruses, we expressed the prediction matrix as $A^{t}=\left\{A_{1}^{t},A_{2}^{t},\ldots ,A_{m}^{t}\right\}$ and represented the equation further by the following matrix form:


(7) $$\begin{array}{l} A^{t+1}=\alpha W_{d}A^{t}+\left(1-\alpha \right).A^{0} \end{array}$$

As t tends to infinity, the expression converges to the following form:


(8) $$\begin{array}{l} A^{*}=\left(1-\alpha \right){\left(I-\alpha W_{d}\right)}^{-1}A^{0} \end{array}$$

where *I*∈*R*^*n*×*n*^is the identity matrix and A^*^ is the association score matrix. For more details on the convergence analysis of label propagation, please refer to the analysis (Wang and Zhang, 2006).

## 3. Results

### 3.1. Experimental setting

In this study, we used 10 times 10-fold cross-validation to evaluate the performance of our proposed WLLP method. Specifically, 90% of the interaction data was used as the training set, and the remainder was used as the test set. For the evaluation results of the 10 prediction matrices, we averaged them. The true positive rate (TPR or recall), false positive rate (FPR), precision, AUC, and area under the precision-recall curve (AUPR) were used as evaluation metrics. The TPR and FPR indicate the ability of the model to correctly predict positive and negative labels. Precision is the ratio of correctly predicted positive labels to all predicted positive labels, and greater precision indicates better prediction performance. The formulas for the TPR, FPR, and precision are as follows:


(9) $$\begin{array}{l} TPR=\frac{TP}{TP+FN}, \end{array}$$

(10) $$\begin{array}{l} FPR=\frac{FP}{TN+FP},\;\text{and} \end{array}$$

(11) $$\begin{array}{l} \text{Precision}=\frac{TP}{TP+FP}, \end{array}$$

where *TP* denotes the number of labels correctly predicted as positive, *TN* denotes the number of labels correctly predicted as negative, *FP* denotes the number of labels incorrectly predicted as positive, and *FN* denotes the number of labels incorrectly predicted as negative.

Area under the receiver operating characteristic curve and AUPR are widely used to evaluate the performance of binary classifiers. We constructed the ROC curve and the precision-recall (PR) curve by calculating the TPR, FPR, and precision. The ROC curve is a probability curve with FPR on the x-axis and TPR on the y-axis at various thresholds (Kumar and Indrayan, 2011; Pegoraro et al., 2021; Sun et al., 2022). The AUC is then the area under the ROC curve, which is primarily used to describe the global prediction performance, where larger values indicate better performance (Tang et al., 2022). An AUC of 1 indicates excellent performance and an AUC of 0.5 indicates stochastic performance (Peng et al., 2020). In addition, the PR curve is more effective than the ROC curve for representing highly unbalanced data, thus we also used the AUPR to fully evaluate the performance of the WLLP model. Similar to the AUC, a larger AUPR corresponds to better prediction performance.

### 3.2. Model comparison

In this study, we compared the WLLP model with four other models, namely SCPMF (Meng et al., 2021), NTSIM (Zhang et al., 2018c), TP-NRWRH (Liu et al., 2016), and VDA-RWR (Peng et al., 2021), for the same HDVD dataset. SCPMF is a drug–virus interaction prediction algorithm based on a novel SCPMF. NTSIM is a drug–disease association prediction method that considers only LNS and label propagation. TP-NRWRH uses the bipartite network projection to enhance similarity and propagates it over a heterogeneous network of drugs and diseases with the help of RWR. VDA-RWR applies RWR to the prediction of the newest drug-coronavirus association.

**Figure 2:**
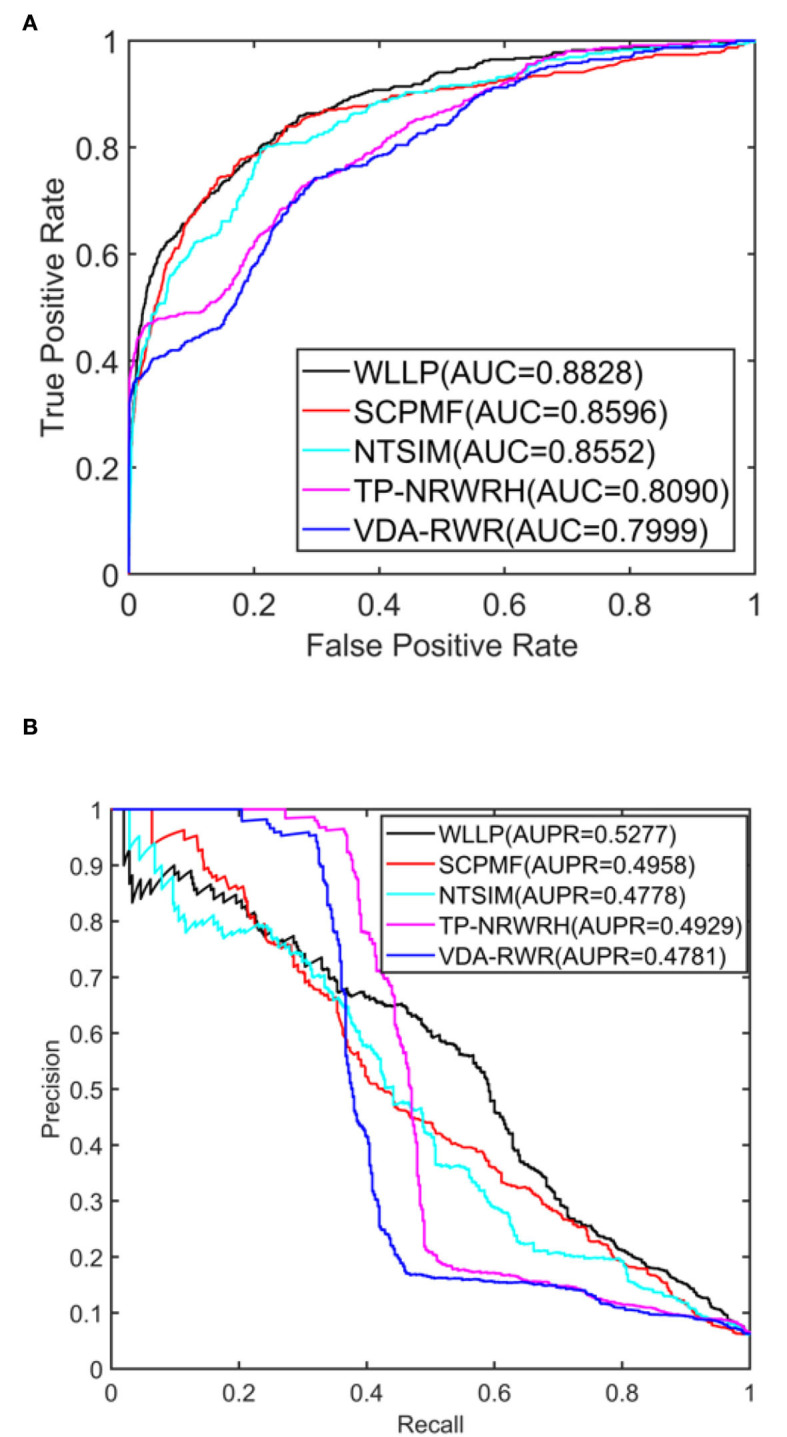
Area under the receiver operating characteristic curve (AUC) and area under the precision-recall curve (AUPR) values of the five prediction methods on the human drug virus database (HDVD) dataset. **(A)** AUC values of the five prediction methods. **(B)** AUPR values of the five prediction methods.

Table 1 shows a comparison of the results obtained from the five prediction models for the HDVD dataset with 10 times 10-fold cross-validation. Figure 2 shows the corresponding ROC and PR curves for the five models. The experimental results demonstrated that the ROC and PR curves of our WLLP model were higher than those of the other four models. It was also apparent that our proposed model offered the best performance in terms of the average AUC and AUPR values. More concretely, the AUC value of WLLP was 0.8828, which was higher than that of the other four approaches (SCPMF: 0.8596; NTSIM: 0.8552; TP-NRWRH: 0.8090; and VDA-RWR: 0.7999). Meanwhile, the AUPR value of WLLP was 0.5277, which was also higher than the other four methods (SCPMF: 0.4958; NTSIM: 0.4778; TP-NRWRH: 0.4929; and VDA-RWR:0.4781). It was not difficult to find that the NTSIM model produced much better results on AUC than the TP-NRWRH and VDA-RWR models, which implied that using LNS was superior to using the original similarity alone, and indicated that using more complex and effective similarity performance provided more important information for association prediction. Due to the effect of the WKNKN pre-training method on the sparsity of the original interaction matrix, the WLLP model produced better prediction results than the NTSIM model, and it also supported the usefulness of the preprocessing procedure (WKNKN) by comparing with the SCPMF model. In summary, the WLLP model exhibited excellent performance.

**Table 1:** Performances of the five prediction methods on the human drug virus database (HDVD) dataset.

**Method**	**10 times 10-fold CV AUC**	**10 times 10-fold CV AUPR**
**WLLP**	**0.8828** **±0.0037**	**0.5277** **±0.0053**
SCPMF	0.8596 ± 0.0011	0.4958 ± 0.0010
NTSIM	0.8552 ± 0.0051	0.4778 ± 0.0110
TP-NRWRH	0.8090 ± 0.0079	0.4929 ± 0.0175
VDA-RWR	0.7999 ± 0.0071	0.4781 ± 0.0143

## 4. Discussion

### 4.1. Ablation experiments

To investigate the plausibility of the WLLP structure, we also tested the model with ablation experiments. We again applied 10 times 10-fold cross-validation to calculate the AUC and AUPR values of the compared models, and the average results were used as the final evaluation indices. The WLLP model comprises three components: WKNKN, LNS, and label propagation (LP). As shown in Table 2, model 1 uses only LNS to set the weights between the nodes on the original label graph and uses label propagation for network diffusion, while model 2 directly applies label propagation to the association network.

**Table 2:** Results of ablation experiments for the weighted reconstruction-based linear label propagation algorithm (WLLP) model.

**Model**	**WKNKN**	**LNS**	**LP**	**10 times 10-fold CV AUC**	**10 times 10-fold CV AUPR**
WLLP	✓	✓	✓	0.8828 ± 0.0037	0.5277 ± 0.0053
Model		✓	✓	0.8552 ± 0.0051	0.4778 ± 0.0110
Model			✓	0.7886 ± 0.0045	0.1028 ± 0.0005

The results presented in Table 2 demonstrated that the WLLP model resulted in better AUC and AUPR values for the HDVD dataset than the other two models. Specifically, for model 1, owing to the sparsity of the original drug–virus association matrix, the lack of diffusion channels without preprocessing using the WKNKN algorithm made the nodes with blank labels received little or no resources during network diffusion, and the propagated information was concentrated on the nodes with high association probability in the global prediction. The introduction of WKNKN alleviated the sparsity of the matrix, and the association prediction of blank labels by WLLP became very simple. Therefore, the WKNKN algorithm can be considered an indispensable part of WLLP. Furthermore, a comparison of model 2 and model 1 clearly revealed that the label propagation algorithm in conjunction with LNS took more information into account than using the chemical structure and sequence similarity alone. The lack of a linear relationship between nodes can make the connections less compact, which in turn leads to poor association prediction for highly unbalanced samples, which is the main reason why the AUPR of model 2 was only 0.1028.

### 4.2. Parameter settings

**Figure 3:**
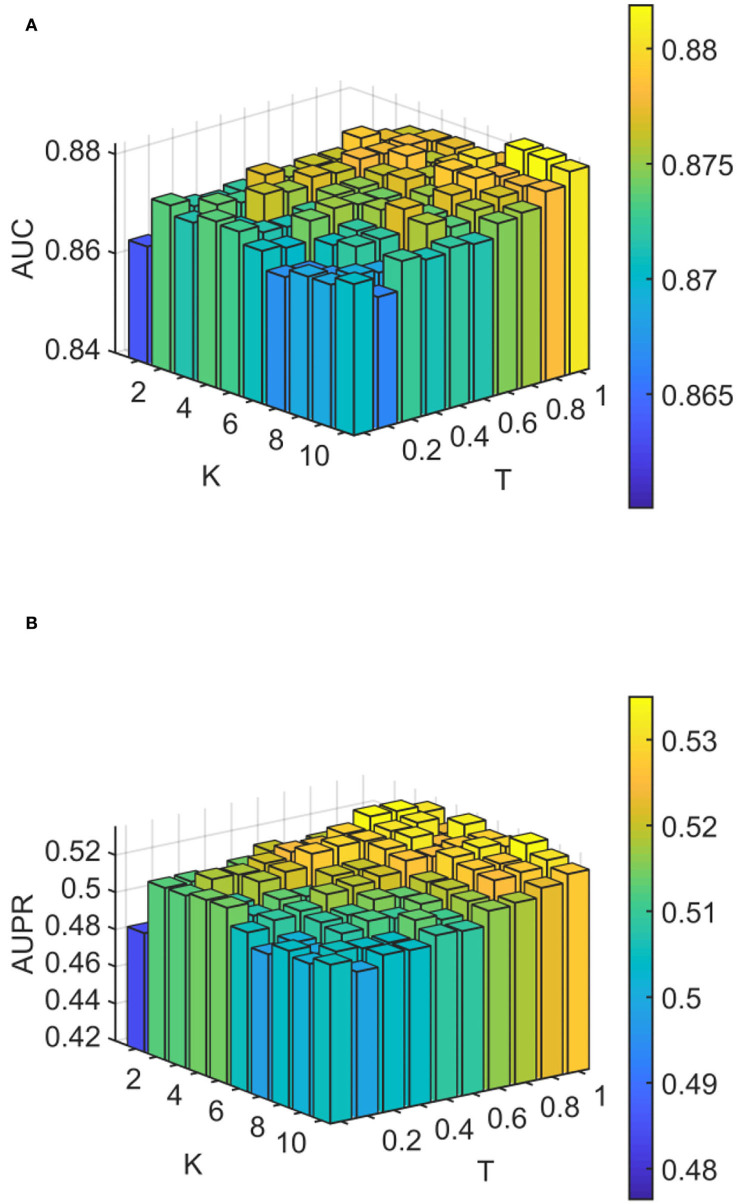
Analytical plots of AUC and AUPR for *K* and *T* in the weighted K-nearest known neighbors (WKNKN) algorithm. **(A)** Analytical plots of AUC for *K* and *T*. **(B)** Analytical plots of AUPR for *K* and *T*.

**Figure 4:**
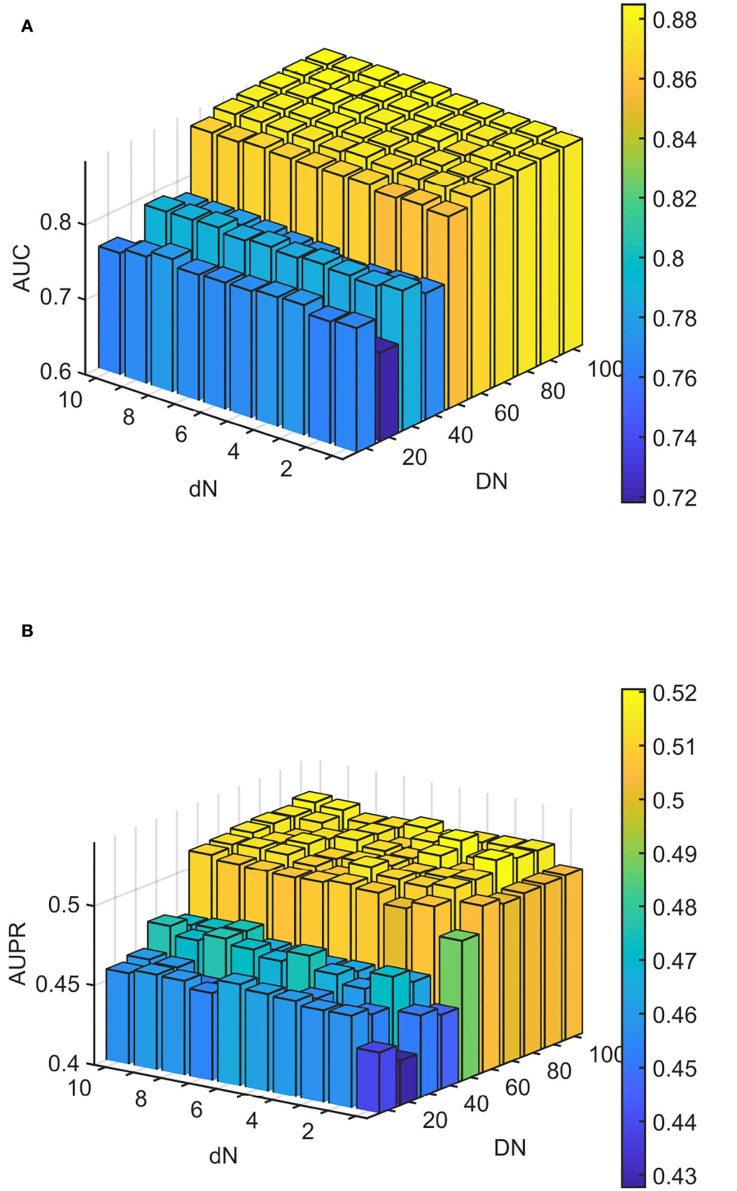
Analytical plots of AUC and AUPR for DN and dN in the linear neighborhood similarity (LNS) algorithm. **(A)** Analytical plots of AUC for *DN* and *dN*. **(B**) Analytical plots of AUPR for *DN* and *dN*.

**Figure 5:**
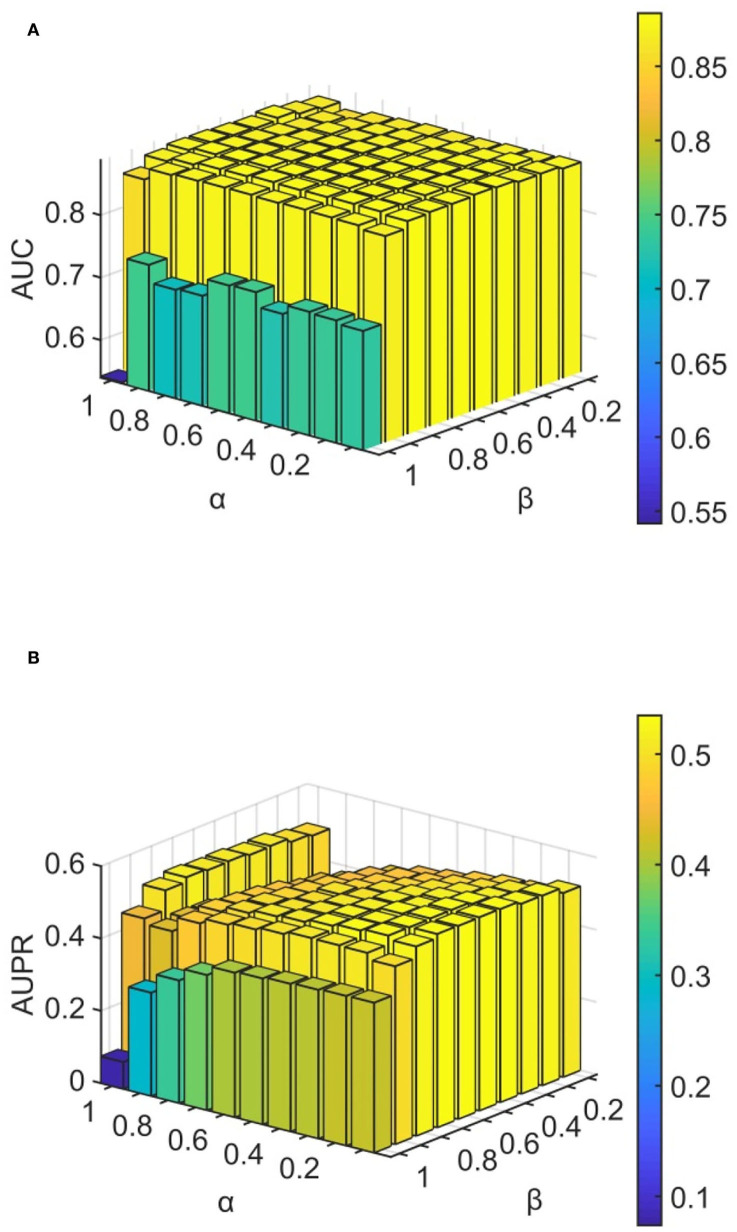
Analytical plots of AUC and AUPR for α and β in the LP algorithm. **(A)** Analytical plots of AUC for α and β. **(B)** Analytical plots of AUPR for α and β.

**Figure 6:**
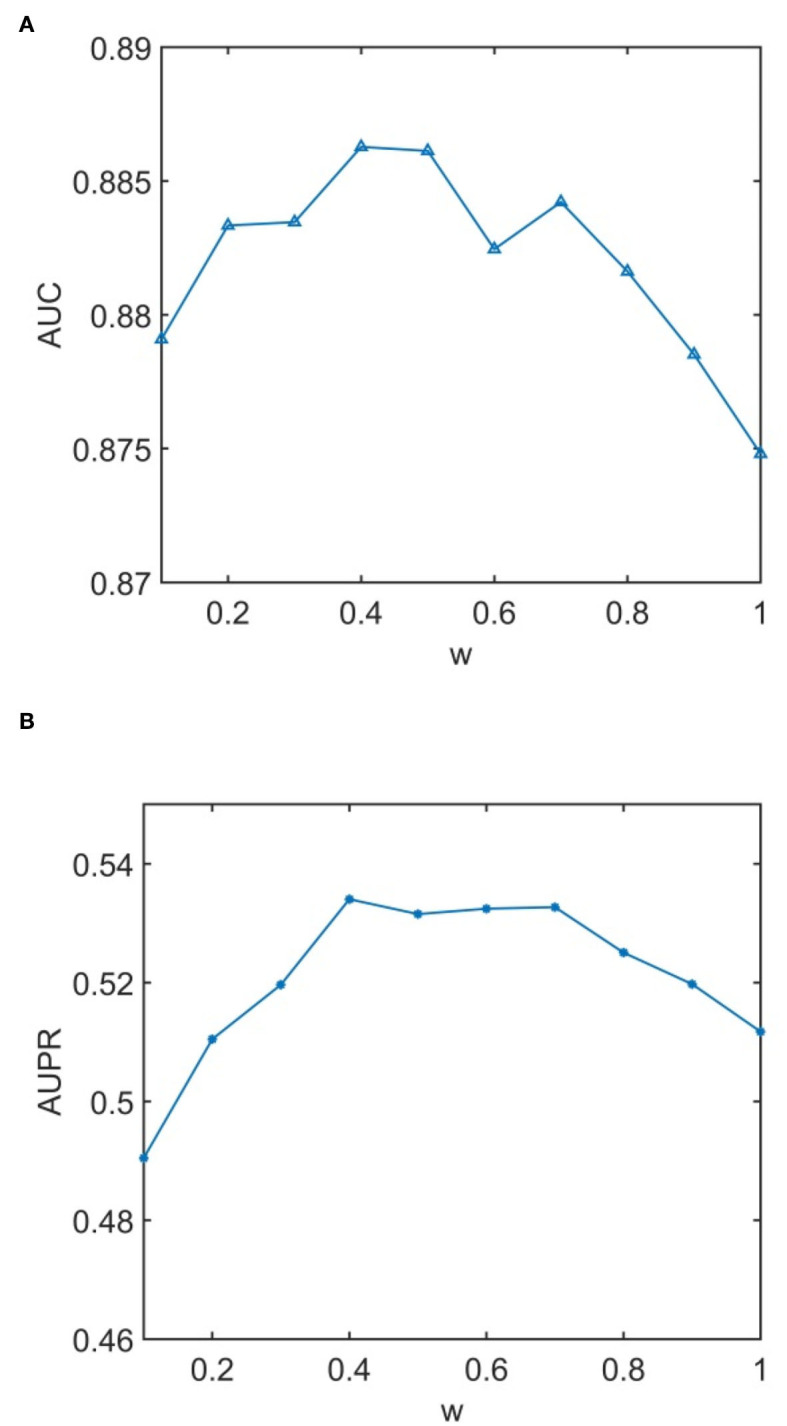
Analytical plots of AUC and AUPR for *w* in the label propagation (LP) algorithm. **(A)** Analytical plots of AUC for *w*. **(B)** Analytical plots of AUPR for *w*.

We conducted experiments to analyze the effect of parameters on model WLLP. To determine the optimal combination of parameters, we used the grid search method. The WLLP model used seven parameters, namely *K*, *T*, *DN*, *dN*, α, β, and *w*, where *K* and *T* are the parameters appearing in the WKNKN algorithm. *K* denotes the maximum neighborhood value in the KNN function, while *T* denotes the decay factor. The adjustment range of parameter *K* is from 1 to 10, while the adjustment range of parameter *T* is from 1 to 0.1. We end up with *K* set to 8 and *T* set to 1 (Figure 3). *DN* and *dN* correspond to the number of elements in the set of nearest neighbors for the drugs and viruses in the LNS calculation process. The number of drug neighbors *DN* should be less than the number of all drugs, and the same is true for the number of virus neighbors *dN* based on previous experience (Chen et al., 2021b). We varied the values from 10 to 100, increasing by 10 each time. In Figure 4, for the label propagation algorithm, we used α and β to represent the retention probability of the update status for drugs and viruses. Thus, we set the different values of α and β from 0.1 to 1 with step 0.1 (Figure 5). Meanwhile, *w* is the label fusion parameter for the final matrix from 0.9 to 0.1 with step 0.1. The effect of the parameter selection of *w* is shown in Figure 6, where we observed that good performance is achieved at *w* = 0.4. The optimal parameter values for the best model performance were found to be as follows: *K* = 8, *T* = 1, *DN* = 100, *dN* = 6, α = 0.2, β = 0.5, and *w* = 0.4.

### 4.3. Case study

The overall aim of this work was to identify possible clues for the treatment of COVID-19 after confirming the performance of the WLLP model. Table 3 lists the top 15 drugs predicted from the HDVD dataset, showing the ranking, drug name, DrugBank ID, and literature evidence for each drug. It can be observed that a majority (80%) of the predicted drugs were supported by a variety of literature evidence. Ribavirin was initially recommended for clinical use in China 2019-nCoV Pneumonia Treatment Plan Version 5-Revised (Khalili et al., 2020). It is the eight predicted drug candidate for the potential treatment of COVID-19. Remdesivir is a nucleotide analog precursor drug with a broad viral spectrum that includes filoviruses, pneumoviruses, parvoviruses, and coronaviruses (Al-Tawfiq et al., 2020; Grein et al., 2020). Remdesivir inhibits viral RNA polymerase and displays *in vitro* activity against COVID-19 (Al-Tawfiq et al., 2020; De Wit et al., 2020; Grein et al., 2020). The combination of remdesivir with emetine may provide better clinical efficacy (Touret and de Lamballerie, 2020). Chloroquine is an inexpensive, safe, and widely administered antimalarial drug that has been used for more than 70 years and is very effective in controlling COVID-19 infection *in vitro* and therefore may be used for the clinical treatment of COVID-19 (Choy et al., 2020). The combination of chloroquine and remdesivir was reported to be very effective in controlling COVID-19 infection *in vitro* (Wang et al., 2020). Based on their combined pathophysiological and pharmacological potential, camostat and nitazoxanide may be recommended for early evaluation and clinical trials against COVID-19 (Khatri and Mago, 2020). Another study provided preliminary evidence for the use of favipiravir in the treatment of SARS-CoV-2 infection (Cai et al., 2020). Umifenovir is a broad-spectrum antiviral drug. In recent years, clinical trials of umifenovir have been initiated in China (O'Boyle et al., 2011). Sodium lauryl sulfate, an anionic surfactant with protein denaturing ability, effectively inhibits the infectivity of several enveloped viruses through denaturation of the viral envelope. Mouthwash containing sodium lauryl sulfate may be effective in preventing SARS-CoV-2 infection through the oral cavity (Sawa et al., 2021). The 18-kDa cytoplasmic protein procyclin A is an important cellular biomolecule required for RNA virus replication, and recent studies have shown that non-immunosuppressive analogs, such as alisporivir, inhibit the activity of procyclins (Almasi and Mohammadipanah, 2020). Saracatinib, sirolimus, and suramin have also been indicated as therapeutic agents for COVID-19 in recent studies (Romanelli and Mascolo, 2020; Salgado-Benvindo et al., 2020; Tatar et al., 2021).

**Table 3:** Top 15 drugs predicted from the HDVD dataset.

**Rank**	**Drug name (DrugBank ID)**	**Evidence**
1	Chloroquine (DB00608)	Choy et al., 2020
2	Hexachlorophene (DB00756)	Unknown
3	Nitazoxanide (DB00507)	Khatri and Mago, 2020
4	Rifamycin (DB11753)	Unknown
5	Remdesivir (DB14761)	Al-Tawfiq et al., 2020; Grein et al., 2020; Meng et al., 2021
6	Odium lauryl sulfate (DB00815)	Sawa et al., 2021
7	Camostat (DB13729)	Zhou et al., 2015; Hoffmann et al., 2020
8	Ribavirin (DB00811)	Khalili et al., 2020
9	Saracatinib (DB11805)	Tatar et al., 2021
10	Alisporivir (DB12139)	Almasi and Mohammadipanah, 2020
11	Tacrolimus (DB00864)	Unknown
12	Favipiravir (DB12466)	Cai et al., 2020
13	Sirolimus (DB00877)	Romanelli and Mascolo, 2020
14	Suramin (DB04786)	Salgado-Benvindo et al., 2020
15	Umifenovir (DB13609)	McKee et al., 2020

For hexachlorophene, rifamycin, and tacrolimus, there are no studies proving their activity against COVID-19. However, hexachlorophene is a common detergent additive used for hand washing and disinfection, while rifamycin is an anti-tuberculosis agent that exhibits antiviral properties against various infectious viruses. Tacrolimus, an immunosuppressant, is commonly used in immunotherapy. Although no studies have been conducted to demonstrate the efficacy of these three drugs against COVID-19, they still have considerable potential, which remains to be further validated by subsequent work of drug developers.

## 5. Summary

To prevent the spread of SARS-CoV-2, it is critical to deepening our understanding of the association between the virus, target proteins, and potential drugs. In the short term, it may be unrealistic to rely on conventional laboratory techniques to develop new drugs against COVID-19, and drug repositioning may represent a more powerful approach. Drug repositioning provides an effective method for prioritizing chemical agents associated with SARS-CoV-2. In this study, a WLLP approach was used to predict the relevance of unknown associations based on drug-virus heterogeneous association networks by combining LNS with LP. The algorithm performs LP on the drug–virus association network, the drug–drug LNS network, and the virus–virus LNS network to diffuse the existing information. With 10 times 10-fold cross-validation, our model achieved an AUC of 0.8828 and an AUPR of 0.5277, both of which were higher than the other methods used for comparison. Furthermore, the information and feasibility of the first 15 drugs were determined by a case study of SARS-CoV-2. Even so, our model still has room for improvement. The predictive performance of the proposed method is limited owing to the current scarcity of data. In the future, we will attempt to tap into drug library and pharmacological resources, and with the addition and integration of more data from recent studies, the prediction results of our model should be improved.

## Data availability statement

The original contributions presented in the study are included in the article/supplementary material, further inquiries can be directed to the corresponding author/s.

## Author contributions

LC and DL: conceptualization, investigation, and project administration. LC: data curation and resources. LC, DL, HX, JL, and LL: formal analysis, funding acquisition, and writing—review and editing. LC, DL, and HX: methodology. HX, JL, and LL: supervision. HX and JL: validation and writing draft. JL: visualization. All authors contributed to the article and approved the submitted version.

## Funding

This work was supported by the National Natural Science Foundation of China (72001202 and 62002070), the Opening Project of Guangdong Province Key Laboratory of Computational Science at Sun Yat-sen University (2021013), and the Science and Technology Plan Project of Guangzhou City (202102021236).

## Conflict of interest

The authors declare that the research was conducted in the absence of any commercial or financial relationships that could be construed as a potential conflict of interest.

## Publisher's note

All claims expressed in this article are solely those of the authors and do not necessarily represent those of their affiliated organizations, or those of the publisher, the editors and the reviewers. Any product that may be evaluated in this article, or claim that may be made by its manufacturer, is not guaranteed or endorsed by the publisher.
